# Strategies and recommendations for embedding sustainability in innovation and design processes

**DOI:** 10.1038/s41598-026-42854-9

**Published:** 2026-03-07

**Authors:** Laura Höpfl, Pascal Dolezalek, Camaren Peter, Alexander Brem, Maria Wirzberger

**Affiliations:** 1 Teaching and Learning with Intelligent Systems, University of Stuttgart, 70174 Stuttgart, Germany; 2 Robert Bosch GmbH, 70469 Stuttgart, Germany; 3 Stuttgart Center of Simulation Science, University of Stuttgart, 70569 Stuttgart, Germany; 4 Graduate School of Business, University of Capetown, Cape Town, 8002 South Africa; 5 Entrepreneurship and Innovation Science, University of Stuttgart, 70569 Stuttgart, Germany; 6 Interchange Forum for Reflecting on Intelligent Systems, University of Stuttgart, 70569 Stuttgart, Germany

**Keywords:** Innovation and design process, Scope 3 emissions, Upscaling, Barriers in the real world, Design for sustainability, Sustainable supply chains, Energy and society, Environmental economics, Psychology and behaviour, Sustainability

## Abstract

The current environmental issues we face are largely due to harmful economic practices. Designing sustainable products and services is crucial for reducing future emissions. Hence, adopting system-oriented models is is essential for encouraging a rethink of current economic and consumption patterns. One important aspect of these approaches is the behavior of consumers, which can be influenced through sustainable product design. For instance, altering the default option to a sustainable alternative can stimulate more environmentally friendly choices by ensuring accessibility and enhancing consumer appeal. However, implementing such design elements requires new structures for the design and innovation process. Therefore, it is important to investigate the most effective design elements for changing behavior and to consider the processes that incorporate these elements. This research is crucial for guiding the development of sustainable product design strategies to address environmental challenges effectively. In our study, we conducted semi-structured interviews (*n* = 6) with industry experts to gain insights into the barriers and motivators of innovation and design for sustainable behavior. Building on these insights, we carried out a quantitative survey with a larger sample of industry experts (*n* = 79) to delve deeper into the identified topics such as process integration or lack of knowledge. Results highlight the importance of integrating sustainability considerations into design and innovation processes to promote sustainable outcomes. Companies are increasingly building internal sustainability structures, yet gaps remain in the use of behavioral interventions such as incentives and choice architecture. Effective strategies, such as training designers and innovators in behavioral change techniques and improving existing processes and guidelines, are crucial. Practitioners favor early and continuous integration of sustainability initiatives. Overall, the results underscore the need to embed sustainability and behavioral insights systematically to support long-term environmental and social responsibility.

## Introduction

The Earth’s system is under significant pressure due to exceeding planetary boundaries^1^ including climate change, biodiversity loss and pollution caused by human activity. In the Anthropocene era, humans substantially influence geological and ecological processes, marking a significant shift in the Earth’s system dynamics^2^. A widely recognized beginning of the Anthropocene is the Great Acceleration in the mid-20th century, which commenced after the second industrialization in Europe^3^. This period was characterized by mass production and increased consumption patterns, heavily reliant on resource extraction and electricity usage^4^. Consequently, the economy transitioned from being rooted in natural cycles to a more linear model, also referred to as a take-make-use-dispose economy^5^. The social and environmental costs of burning fossil fuels and losing biodiversity were neglected through this shift of economic systems.

Given that challenges like climate change have systemic origins, they are more effectively addressed through systemic solutions than isolated solutions^6–8^. Iacovidou et al.^9^ emphasize a range of strategies to promote systemic thinking and modeling for sustainability. In this context, the authors also discuss resource recovery systems such as cradle-to-cradle, which advocates for a fully integrated circular economy (CE)^10^, as well as the performance economy, which involves offering goods as services through rental, leasing, and sharing models. In this model, manufacturers retain ownership and responsibility for risks, waste^11^, and pollution, aligning closely with the principles underlying the CE. Circular economy mimics natural life cycles^5^ and therefore has fewer adverse consequences for the planet, not only in terms of biodiversity loss and greenhouse gas emissions but also through the reduction, substitution, or traceability of hazardous substances. By promoting closed material loops in which harmful substances are identified, minimized, or replaced, CE practices mitigate both GHG and pollutant emissions, countering the predominantly linear economies of modern times^12^.

Iacovidou et al.^9^ postulate five interconnected subsystems that need to be considered for supporting transitions to CE: (1) resource flows and provisioning service; (2) governance, regulatory framework, and political landscape; (3) business activities and the market; (4) infrastructure and innovation; and (5) user practices. Bocken et al.^13^ summarize several systems design strategies that could facilitate shifting to a CE, such as slowing and closing resource loops and reducing resource flows: Through design for product-life extension, the resource loops can be slowed down by, for instance, the ease of maintenance, repair, and upgradability. The loop between post-use and production can be closed through recycling, for example, resulting in a circular flow of resources^13^. Lastly, resource efficiency and narrowing resource flows aim to reduce the resource flow per product^13^. Furthermore, CE needs to consider socio-metabolic flows, which extend beyond resource flows and encompass the movement of various resources such as data, money, and people. The CE needs to consider these dynamics to fully address the diverse pressures and dynamics, rather than solely focusing on resource flows^14,15^.

In the context of a CE, it is essential to analyze the complete supply chain. The GHG Protocol is a widely accepted standard outlining the methodology for assessing direct emissions (Scope 1), emissions linked to the supply of electricity, heating, and cooling (Scope 2), and upstream and downstream value-chain emissions not directly associated with the purchase of energy (Scope 3)^16,17^. Scope 3 downstream emissions encompass a wide range of indirect emissions, including those generated by the use of products and services by customers, such as the consumption of fuel from vehicles or electricity from buildings. Additionally, emissions resulting from the disposal of products and waste, such as the decomposition of organic waste in landfills, are also considered part of Scope 3. Product emissions vary across different scopes, but due to rising greenhouse gas emissions at the end consumer level, Scope 3 is becoming increasingly important^18^. We focus on Scope 3 emissions, as these for many companies outweigh the emissions from the other scopes^19^. Unlike Scope 1 and 2, Scope 3 emissions are largely beyond the direct control of the company, but are crucial for a complete view of product-related environmental impacts. Given the growing importance of global value chains and rising product-related greenhouse gas emissions, Scope 3 is becoming increasingly relevant^18^.

During the Industrial Revolution, designers and engineers played a crucial role in promoting a linear economy by implementing strategies like planned obsolescence. This concept aimed to boost economic growth by artificially encouraging excessive consumption in the market, ultimately contributing to the recovery from the Great Depression in the 1930s^5^. Planned obsolescence is the outcome of a deliberate decision by suppliers that a product should no longer be functional or desirable after a predetermined period^20^. This example highlights the crucial role of designers and innovators in advancing products that align with the principles of the CE^5^. Similar to their historical counterparts, present-day designers and innovators can substantially influence supply chain sustainability through material usage and product usage behavior. Therefore, their efforts can now be strategically directed toward facilitating the transition to a CE, thereby contributing to the reduction of emissions. For example, analyzing and integrating the behavioral impacts associated with their products can provide valuable insights into emission reduction strategies^21^. Emissions can be mitigated through targeted behavioral interventions, such as implementing defaults, providing feedback, and leveraging social modeling^22^. For example, Tang and Bhamra^23^ described seven intervention strategies designers can employ: eco-information, eco-choice, eco-feedback, eco-spur, eco-steer, eco-technical intervention, and clever design. These strategies could be utilized in the innovation of new products and services, especially for enhanced sustainability use, the (re)design of new products, or incrementally improving the sustainability of a product. In the context of innovation and design, implementing sustainable design requires expanding existing process models to integrate sustainability as a core element^24^. Existing innovation processes focus on desirability, viability, and feasibility^25^. However, only a few incorporate sustainability as a fourth dimension.

We identified significant gaps in current research and practical understanding. The increasing importance of downstream Scope 3 emissions underscores the urgent need to apply knowledge about sustainable design and innovation in real-world contexts. Addressing these emissions requires enhancing the accessibility, convenience, and desirability of sustainable choices for individuals through innovative design. Therefore, there is a critical necessity for collaboration between researchers and practitioners in the fields of design and innovation to advance sustainability in practical applications. Furthermore, it is imperative to investigate the essential conditions for developing products that encourage sustainable user behaviors. Hence, integrating sustainability principles into the design and innovation processes should specifically target emissions directly associated with user behavior (Scope 3). Additionally, finding ways to prioritize innovation and design strategies for behavioral change can create effective, appealing solutions that promote sustainable behaviors on a broader scale. Building on this perspective, the present study draws on the CREATE behavioral design framework^26^. CREATE conceptualizes behavior as a process involving Cue, Reaction, Evaluation, Ability, Timing, and Experience. Design features and behavioral interventions can influence one or more of these stages. This framework guided the mixed-methods design of the study by linking design decisions, behavioral mechanisms, and sustainability-related outcomes.

Consequently, the research questions emerge, (1) how innovation and product development processes can be adapted so that users act more sustainably in the use phase? And (2) at what point in the innovation and design process should a sustainable innovation or intervention be implemented to be most impactful? To address these questions, our research explores how design and innovation can effectively promote sustainability, utilizing the potential of innovation, design, and behavioral interventions to reduce emissions as a result. Our primary objective is to facilitate a more sustainable use phase for the end consumer, thereby contributing to a more sustainable supply chain. Therefore, optimizing both production processes and the customer’s use phase is necessary. We concentrate on changed consumer behavior enabled by innovation and design practices, analyzing how to translate their potential into actual emission reduction. Our goal is to provide guidance that helps to analyze real-world problems closer and, therefore, helps the industry and its designers strongly incorporate sustainability in their future products.

We employed a mixed-methods approach to investigate the use of product features that promote sustainability and behavioral interventions. Semi-structured interviews were first conducted with six purposively selected innovators and designers to obtain in-depth insights into sustainable design strategies and behavioral interventions, such as feedback and default options. The qualitative findings informed the development of the questionnaire for the subsequent quantitative phase. In this phase, the identified themes were examined in a larger sample of 79 participants to assess their prevalence and capture the status quo.

From our results we derive guidelines for an adapted process for sustainable interventions and design (Appendix 1). Adapting existing processes and implementing effective strategies, such as providing training in behavioral change interventions for designers and innovators, are crucial for enhancing sustainability initiatives and fostering long-term environmental and social responsibility. Based on the study’s outcomes, we emphasize the importance of integrating sustainability into the design and innovation process, especially the need for behavioral interventions to reduce emissions and promote circularity.

## Methods

Utilizing both qualitative and quantitative methods of investigation, we examined the potential for adapting innovation and product development processes to encourage more sustainable behavior from users during the use phase. Additionally, we sought to determine the most effective point in the innovation and design process to implement sustainable innovations or interventions for maximum impact. We started by conducting semi-structured interviews (*n* = 6, Part 1) to inform the quantitative portion of the study (*n* = 79, Part 2).

The data collection plan for the study was approved by the Committee for Responsibility in Research at the University of Stuttgart (approval number: Az.24-016). Informed consent was obtained from all participants, and the study adhered to the General Data Protection Regulation (GDPR) from the EU^27^. Additionally, we closely followed the guidelines and regulations outlined in Standard 8 of the Ethical Principles and Code of Conduct for Psychologists^28^.

### Part 1: Interviews

#### Participants

We interviewed six participants about strategies related to sustainable design and innovation. The participants included women (*n* = 5) and men (*n* = 1), between 26 and 42 years ($M_{age}$ = 37.6 years, $SD_{age}$ = 7.44 years). As the study was conducted in collaboration with the Robert Bosch GmbH, participants were primarily recruited through the company’s internal network associated with innovation and design units, as well as through external social media channels. This approach leveraged both internal company resources and external outreach. Participants received no monetary compensation for participating in the interview.

#### Materials

Our research explores three key themes related to sustainable product design and customer behavior. The first theme focuses on developing sustainable products that promote energy efficiency and encourage environmentally friendly choices among users. This research involves understanding and leveraging focus topics such as behavior change, environmental awareness, incentives and rewards, and social norms to drive sustainable product design. The second theme delves into the factors that influence sustainable customer behavior during the use of real products, encompassing topics such as habits, social influences, self-efficacy, environmental awareness, and incentives and rewards. Lastly, the third theme centers on methods and process steps that innovators and product designers can utilize to ensure that products are designed to encourage sustainable behavior, particularly regarding energy efficiency and the reduction of Scope 3 emissions. This theme integrates psychological constructs, including behavior change, incentives and rewards, user-friendliness, social norms, and environmental awareness, to guide the design process toward sustainability.

#### Data collection and preparation

The study was conducted using semi-structured interviews to explore participants’ experiences with sustainable product design and innovation. Participants for the expert interviews were purposively selected based on their professional role and expertise in sustainable innovation and design. They were identified and contacted through social media and Bosch’s internal innovation and design networks. Interviews were conducted remotely utilizing an online collaboration tool to enable participation and ensure privacy. Each session lasted approximately 65 minutes and was audio-recorded with the participant’s consent. A semi-structured interview guide was utilized to ensure consistency across sessions while allowing flexibility to explore relevant topics introduced by participants. Participants were first asked general questions about their roles and responsibilities at work, followed by more focused questions about their experiences with product design, innovation and sustainability. All interviews were transcribed verbatim for subsequent thematic analysis and tagged with a program for qualitative data analysis.

#### Data exploration

On average, interviews lasted for $M_{min}$ = 65.4 min ($SD_{min}$ = 8.34 min). Reflexive thematic analysis (RTA) was employed for data analysis, using an inductive approach to interpret the qualitative dataset^29^. The analysis involved revisiting the interview transcripts to identify overarching themes across participants. Relevant excerpts were gathered, reviewed, and categorized by two independent raters. We carried out a systematic and iterative examination of the transcripts and their corresponding categories, aligning the relevant excerpts with the Bosch Innovation process^30^ to pinpoint research questions for the second phase.

### Part 2: Survey

#### Participants

Participants were recruited via targeted invitations through social media and Bosch’s internal innovation and design networks. External recruitment channels included specialized networks on social media such as the Behavioral Science in Environment (Bescy) group^31^ and the Sustainable User Experience (SUX) network^32^. Participants received no monetary compensation for participating in the interview. The sample consisted of 79 individuals holding various roles, including sustainability manager (*n* = 15), innovator (*n* = 15), consultant (*n* = 9), product manager (*n* = 9), user researcher (*n* = 7), designer (*n* = 5), IT professional (*n* = 2), executive (*n* = 1), dedicated behavioral researcher (*n* = 1) and others (*n* = 15). Most participants were employed in large companies with more than 500 employees (*n* = 59) or did not provide this information (*n* = 20).

#### Materials

The survey questions were formulated based on the findings of the initial phase of the study and relevant literature^26^. The topics covered in the questions encompass the development of sustainable products, utilizing behavioral intervention techniques for influencing user behavior, the optimal stage in the innovation process for receiving information on behavioral interventions, and identifying best practices or methods for promoting sustainable behavior change. Sample questions include inquiries about the company’s efforts in developing products that encourage sustainable behavior among consumers, the perceived obstacles in integrating sustainability into the innovation process, the specific behavioral intervention techniques employed to influence user behavior, and the preferred timing for receiving information and reminders about behavioral interventions during the innovation process. Detailed guidelines for the survey can be accessed in the supplementary material provided at https://osf.io/bt5ha, offering comprehensive insights into the research methodology.

#### Data collection and preparation

Participants completed the study individually and remotely, accessing the survey tool SoSciSurvey^33^ via social media platforms or internal communication channels. Upon providing informed consent, participants were presented with 16 standardized questions, which took approximately 5-10 minutes to complete. Following the survey, participants received a comprehensive debriefing regarding the study’s objectives and were given the option to opt-in to receive the results via email.

## Results

### Part 1. Qualitative results

Upon conducting a rigorous qualitative thematic analysis^29^ of the interview data, several pivotal factors emerged as critical determinants of sustainability integration. One such factor was the apparent lack of knowledge and understanding regarding behavioral change interventions, highlighting a potential gap in the understanding of how to effectively drive and sustain behavioral changes that align with sustainable practices. Additionally, the analysis revealed diverse measures that have been implemented within organizations to foster sustainability, ranging from policy changes to the introduction of new technologies and processes. Furthermore, the interviews unveiled the multifaceted obstacles faced in sustainability integration, encompassing organizational, cultural, and operational challenges that hinder the seamless incorporation of sustainable practices into innovation processes. Lastly, the significance of anchoring sustainability in the innovation development process and innovation process emerged as a crucial theme, emphasizing the need to embed sustainability considerations at the core of the innovation life-cycle. These findings collectively underscore the intricate and multifaceted nature of sustainability integration within innovation processes, pointing towards the need for holistic and strategic approaches to address these challenges effectively. In Table 1 themes evolving from interviews with excerpts are shown. A comprehensive list of quotes is provided in the supplementary materials (https://osf.io/bt5ha/).

**Table 1:** Themes evolving from interviews with excerpts.

Themes	Excerpts
Scaling	“There are different points, projects, and then there are, of course, the processes and initiatives from the strategic level to the operational level, in the implementation phase.”
Strategy	“To develop in terms of technology (...) is much more of a strategic and programmatic exercise.”
Desirability and feasibility	“The biggest lever now (...) is to care for what you love, so to speak, to design products that are important and that you might also form an emotional bond with”
Obstacles in sustainability integration	“Anchoring sustainability in the innovation development process and innovation process (...) must be easy to understand and design must be integrated. So sustainability must not be considered in parallel or additionally”
Lack of knowledge in behavioral interventions	“Behavior makes such a difference and we can actually influence it relatively easily. Why don’t we do it?”
Measures implemented to foster sustainability	“Now it’s about coming up with a strategic approach: The Circular Economy (...). We take these models, that try to recover the materials a little more”

### Part 2. Quantitative results

Related to the adoption of sustainable products and development of products that actively encourage sustainable behavior among consumers by their respective companies, participants answered ‘yes, most products’ (18.18%), ‘yes, some products’ (74.03%), ‘no’ (6.49%), and ‘I do not know’ (1.3%).

**Fig. 1:**
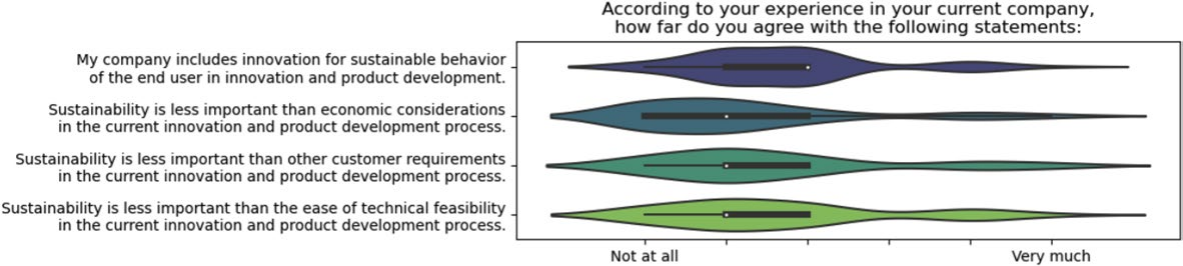
Participants’ perspectives on the significance of sustainability within their respective organizations.

In Fig. 1, four statements depict the participants’ perspectives on the significance of sustainability within their respective organizations compared to other factors such as economic considerations, customer requirements, and technological feasibility. The majority of respondents expressed moderate agreement with the statement, ‘my company incorporates innovation for sustainable behavior of the consumer in innovation and product development,’ indicating a central tendency towards agreement but not universal consensus. Responses to the statement ‘sustainability is less important than economic considerations in the current innovation and product development process’ exhibited the widest spread, suggesting variability in how companies prioritize sustainability in relation to economic factors. The statement ‘sustainability is less important than other customer requirements in the current innovation and product development process’ garnered responses that skewed towards agreement, indicating that customer requirements often precede sustainability in decision-making. The statement ‘sustainability is less important than the ease of technical feasibility in the current innovation and product development process’ received the most robust agreement from respondents, highlighting that technical feasibility is often prioritized over sustainability.

When queried about the specific actions that have been put in place to advance sustainability in product development, participants responded with the implementation of ‘trainings in sustainability’ (68.35%), ‘internal company guidelines on sustainability’ (64.56%), ‘sustainability reporting’ (62.03%), ‘KPIs for sustainability’ (49.37%), ‘sustainability officers’ (49.37%), ‘sustainable guidelines for suppliers’ (45.57%), ‘criteria for sustainability in processes (e.g., innovation or development processes, quality gates’ (39.24%), ‘criteria for sustainability incentivization systems’ (3.8%), ‘other’ (5.06%), and ‘none’ (6.33%).

Regarding the existence of sustainability criteria and guidelines within their company’s innovation and product development processes, participants affirmed the presence of such measures by responding with a resounding ‘yes’ (64.56%), ‘no’ (10.13%), and ‘I do not know’ (22.78%).

When asked what types of behavioral intervention techniques participants have already utilized, they answered ‘not working in this domain’ (49.37%), ‘attention’ (32.91%), ‘social interventions’ (25.32%), ‘habit formation’ (20.25%), ‘organizational architecture’ (16.46%), ‘choice set’ (10.13%), ‘incentivizing’ (10.13%), ‘friction’ (5.06%), and ‘none’ (5.06%).

Referring to potential obstacles encountered in integrating sustainability into the innovation process, participants reported, among others, the ‘perception that sustainability adds costs’ (64.56%), the ‘lower priority of sustainability compared to other criteria’ (62.03%), ‘lack of commitment for long long-term success on sustainability by leadership’ (55.70%), ‘lack of investment of resources into sustainability’ (54.43%), and ‘resistance of stakeholders, e.g., prioritizing shorted term profits’ (53.16%), ‘difficulties in complementing sustainability due to complexity of the topic’ (30.81%), ‘lack of shift in the mindset towards sustainability’ (28.44%), ‘lack of knowledge of how to increase sustainability’ (26%), ‘lack of awareness within the organization’ (22.12%), ‘difficulties in implementing sustainability due to technological challenges’ (16.59%), ‘lack of criteria for sustainability’ (16.59%), ‘other’ (2.37%).

On the matter of including sustainability in decision criteria, KPIs, or metrics would promote sustainable product development, most participants stated ‘Yes, all of them would help’ (60.76%), ‘Yes, KPIs would help’ (11.39%), participants ‘do not know’ (11.39%), ‘Yes decision criteria would help’ (7.59%), ‘Yes, metrics would help’ (5.06%), ‘No’ (1.27%).

Asked if guidelines for product development and innovation taking into account sustainability would be helpful, participants reported that the respective companies already ‘have guidelines, which could be improved’ (35.44%), ‘yes’ (49.37%, participants ‘do not know’ (6.33%), ‘no’ (6.33%).

Regarding the question at what points in the innovation process it would be helpful to receive information and reminders about behavioral interventions, most participants answered ‘through the whole process’ (63.29%), ‘in the beginning of the innovation process’ (41.77%), ‘in the concept-ideation step’ (32.91%). Asked if the point of behavioral interventions would differ for sustainability interventions, participants stated ‘no’ (39.24%), ‘do not know’ (36.71%), ‘yes’ (17.72%), and ‘other’ (2.53%).

Taken together, the results show that companies increasingly establish internal structures such as sustainability trainings, internal guidelines, and sustainability KPIs, with regulatory frameworks such as the EU CSRD contributing to this development. The data also indicate shortcomings in sustainable supplier criteria, in incentives for sustainable practices, and in the use of behavioral interventions, particularly choice architecture and incentive mechanisms. Although participants rated sustainability as highly important, the practical implementation of sustainable design measures remains limited. Regarding the timing of sustainability interventions within the innovation process, practitioners reported a preference for integration across all phases, with emphasis on early stages. Overall, the findings indicate that sustainability-related and behavioral elements are not yet consistently or systematically embedded in innovation and design processes.

## Discussion

The study aims to explore the impact of sustainable product design on consumer behavior and develop effective strategies for promoting sustainable design and innovation. We included interviews and a quantitative survey with industry experts to identify barriers and motivators of sustainable behavior. Our goal is to present recommendations to enhance sustainability initiatives and foster long-term environmental and social responsibility.

### Key results

The analysis yields valuable insights directly relevant to our first research question, which investigates strategies for adapting innovation and product development processes to promote more sustainable user behavior during the use phase. In general, the study revealed that organizations have taken significant steps in building internal capacity and accountability for sustainable practices, such as implementing sustainability trainings, internal company guidelines, and sustainability reporting. The inclusion of sustainability KPIs and dedicated sustainability officers further demonstrates a growing trend toward integrating sustainability metrics and roles within product development processes. The growing importance of KPIs and reporting in the field of sustainability is possibly influenced by regulations such as the Corporate Sustainability Reporting Directive (CSRD) from the European Union^34^. Our data supports this notion as it indicates a growing impact of regulatory frameworks on organizational sustainability practices. Another potential benefit of incorporating sustainability KPIs within the corporate strategy is achieving strategic alignment to gain competitive advantage^35^.

The existing data also indicates specific areas that require improvement, especially in the implementation of sustainable guidelines and criteria for suppliers, which has been a topic of discussion in literature in the past^36^. Additionally, we found limited focus on incentivizing sustainable practices in product development, even if already discussed in the literature and applied by frameworks^37,38^. These findings highlight the need for organizations to broaden their sustainability initiatives beyond internal capacity building and reporting to encompass external stakeholders and product development processes. An user-centered, more individualized approach is by utilizing user clusters through targeted incentives^39^.

The study also revealed that participants see significant potential in more closely integrating sustainability into the innovation and design process. Moreover, participants are less involved in behavioral intervention techniques, indicating potential knowledge gaps in this area. While attention, social interventions, and habit formation were more commonly utilized, other techniques, such as choice architecture and incentivizing, were less prevalent. This lack suggests a need for broader implementation and knowledge dissemination to enhance the effectiveness of behavioral interventions, particularly in promoting sustainable behavior among end consumers.

The responses from participants regarding the high absorption of sustainability-oriented products that promote sustainable behavior among end consumers indicate that there is still progress to be made in this area. Existing literature also emphasizes the need for further advancements in the design to promote sustainable behavior among consumers^40^. Participants’ acknowledgment of the importance of sustainability underscores the growing recognition of sustainability as a critical factor in organizational operations. The disparity between the perceived importance of sustainability and its practical implementation, as supported by both empirical data and existing literature, e.g., Brennan et al.^41^, underscores the urgency for more effective strategies to narrow this gap and translate awareness into tangible actions.

In response to our second research question, regarding the optimal timing for implementing sustainable innovations or interventions within the innovation and design process for maximum impact, practitioners preferred integrating these initiatives at all stages. However, the majority leaned towards early integration, recognizing the potential for a more significant impact. In the discussion on integrating behavioral interventions into the innovation process, the findings underscore the importance of continuous support throughout the entire process. The varied responses regarding the timing of sustainability interventions emphasize the need for further exploration of the intersection between behavioral and sustainability interventions within the innovation process, highlighting the importance of considering the timing and integration of these interventions to drive innovation toward sustainable outcomes.

Existing research provides frameworks to bring behavioral insights to the real world^42,43^. White et al.^43^ postulate the SHIFT framework to close the attitude-behavior gap, utilizing social influence, habit formation, the individual self, feelings and cognition, and tangibility. According to Wendel^26^, key obstacles designers and innovators must consider include user distractions, lack of awareness, negative reactions, unfavorable cost–benefit evaluations, limited ability to act at the moment, and low perceived urgency. The analysis reveals that organizations have taken important steps to integrate sustainability into product development through internal capacity building, governance structures, and reporting (e.g., sustainability trainings, KPIs, and dedicated roles). While these measures support the organizational ability to design sustainable products, our findings indicate that behavioral interventions are still underdeveloped in practice. In particular, sustainability efforts frequently focus on internal processes rather than on user-facing design strategies that address key barriers to sustainable behavior. Mapping common behavioral interventions to the CREATE framework^26^ shows that organizations mainly target attention (e.g., labels), while choice architecture and incentives are often neglected. This suggests that the current status quo in innovation and design is more oriented toward internal sustainability governance than toward systematically designing interventions that reduce barriers across all stages of user decision-making.

### Theoretical implications

Enhancing the sustainability of products and services is a critical goal. The significance of the use phase varies across companies, with behavioral interventions being particularly crucial for organizations where energy emissions during the use phase constitute a substantial proportion of their environmental impact. However, both theoretical and practical insights into this area remain limited. This highlights the need for further research to address these gaps and develop effective, evidence-based approaches. Most participants expressed support for integrating sustainability into product development and innovation guidelines, indicating widespread recognition of the potential positive impact of prioritizing sustainability in development and innovation processes. Even though literature portrays the progress of products and innovation guidelines^44–46^, there is a critical need for research aimed at developing and refining guidelines that effectively embed sustainability principles within organizations. Such efforts can foster a culture of sustainability by leveraging and enhancing the innovation process. This research presents an opportunity to explore and identify best practices for integrating sustainability principles into the innovation process and to develop actionable guidelines that organizations can implement. By doing so, researchers can play a pivotal role in driving the adoption of sustainable practices within the innovation process, ultimately contributing to a more sustainable and environmentally conscious business landscape. The guidelines should be grounded in a systematic analysis and incorporate multiple paradigms to ensure the successful implementation of sustainability projects.

The UN Sustainable Development Goals (SDGs) are the foundation for European reporting standards^47–49^. Research should investigate here to specify useful reporting and minimize the negative outcomes. Furthermore, one of the critical challenges identified in the study is the perception that sustainability adds costs, reflecting concerns about financial implications. This perception, coupled with the need for more investment of resources into sustainability and stakeholders’ resistance, poses significant obstacles to integrating sustainability into the innovation process. Supporting this, Bigliardi et al.^50^ identified ‘knowledge,’ ‘collaboration,’ ‘organizational,’ and ‘financial and strategic’ as the four main barriers to innovation in Small and Mid-sized Enterprises (SMEs). These findings underscore the multifaceted nature of the challenges faced in promoting sustainability within innovation, emphasizing the need for research to address not only financial considerations but also leadership commitment, resource allocation, and stakeholder engagement.

### Practical implications

In practice, while stakeholders acknowledge the importance of Scope 3 emissions, they often lack concrete strategies or actionable insights. To promote sustainable behavior, companies should implement systematic methods and tools, adapt design and innovation processes to support sustainable solutions and prioritize closing the knowledge gap between behavioral researchers and designers. Only a small portion of participants reported using behavioral strategies, such as incentivizing sustainable customer behavior or implementing systems that promote sustainability, highlighting a significant area for improvement. Participants demonstrate limited awareness of this gap, indicating a need for increased attention and education on this critical issue. To address this gap, sustainability managers and innovators must be equipped with the knowledge and resources necessary to reduce Scope 3 emissions effectively. To support these efforts, we developed a deliverable in form of guidelines and recommendations. The deliverable provides high-level recommendations on these topics, accompanied by guiding questions designed to be addressed throughout the innovation and design processes (Appendix 1).

Regulatory bodies like the EU are crucial in ensuring the effective implementation of regulations, but it is important to keep desired outcomes in mind and avoid unnecessary bureaucracy. Reporting is essential but should be more strategic to drive real change^51^. Companies should implement systematic methods and tools to foster sustainable behavior, adapt design and innovation processes, bridge the knowledge gap between behavioral researchers and designers, incentivize customer behavior, and allocate resources for reducing Scope 3 emissions. Design-led behavioral interventions can reduce the environmental impact of household products^52^. Systematizing the use of behavioral intervention strategies can foster sustainable consumption behavior^53^.

### Limitations

Limitations of this study include the fact that our findings are primarily applicable to large companies, as the majority of our participants are employed by organizations with over 500 employees. Furthermore, due to the absence of standardized, validated scales, the survey items were self-developed, which may limit comparability with prior research and requires cautious interpretation regarding generalizability. Additionally, the study emphasizes the importance of developing a strategy for improving behavioral uptake with a deliverable. These guidelines are intended to serve as an initial framework, and we encourage both researchers and practitioners to further refine and expand upon them, accompanied by rigorous testing to assess their effectiveness. Furthermore, the existence of social biases introduces uncertainty regarding the extent to which participants favored the socially desirable option^54–56^, in this case pro-sustainability. The partially internal recruitment methods might reinforce this issue. As a result, we are unable to ascertain whether the perceived high importance of sustainability is genuine or merely a reflection of practitioners expressing sentiments they believe are favorable.

### Future perspectives

In future research, it is essential to conduct comprehensive evaluations and comparisons of systematic methods and tools of the development process on a larger scale. Future research should validate and refine the self-developed instrument and test its applicability across different organizational contexts to improve comparability and generalizability. Additionally, there is a need to assess the effectiveness of methods to bridge the knowledge gap among designers and innovators. Furthermore, future research should prioritize investigating strategies to cultivate a shift in mindset among employers toward sustainability. Therefore, practical guidelines should be extracted from theoretical work where possible. This guideline should be further communicated to the public to enable practical appliance and refining of the work. According to Chofreh et al.^57^, sustainability paradigms encompass environmental, social, and economic aspects, as well as decision paradigms that include strategic, tactical, and operational considerations. Investigating whether variations in addressing these considerations result in distinct methods for integrating them into the innovation or design process would provide valuable insights.

Additionally, exploring whether individuals with a sustainable mindset are more effective in integrating sustainability into product design and the extent to which this integration should be anchored would provide valuable insights. As sustainability is maturing from a trend towards becoming a key differentiator, it is essential to understand its increasing significance. Furthermore, exploring critical challenges such as financial limitations and internal organizational dynamics presents a compelling opportunity for further research.

Finally, it should be noted that the focus of this study on Scope 3 emissions could be complemented by a life cycle assessment perspective. This would capture additional environmental impacts beyond climate change and should be considered in future research.

In conclusion, this study provides an overview of current sustainability efforts in organizations, particularly the promotion of sustainable consumer behavior and the integration of sustainability into innovation processes. Despite its increasing relevance, sustainable design is still applied only to a limited extent. Companies are developing internal structures such as training programs, guidelines and sustainability KPIs, driven by regulations like the EU CSRD. However, gaps persist in supplier criteria, incentives and the use of behavioral interventions.

Our results suggest that organizations primarily address users’ attention, for example through labeling. Interventions that influence habits, evaluation, ability, timing, and experience, such as choice architecture and incentives from the CREATE framework, are only rarely utilized. This indicates that current efforts are still largely focused on internal governance rather than on systematically reducing behavioral barriers across all stages of user decision-making.

Training on behavioral change for designers and innovators is likely to be crucial for advancing sustainable design and reducing Scope 3 emissions. Practitioners favor embedding sustainability across all innovation phases, especially at early stages. Overall, the results emphasize the need to integrate sustainability and behavioral insights more systematically and earlier in innovation and design processes to foster long-term environmental and social responsibility.

## Data Availability

The datasets generated during and analyzed during the current study are available in the OSF repository, https://osf.io/bt5ha/.
